# The genetic diversity of the genus Mus (Linnaeus, 1758) in the eastern part of the North Caucasus

**DOI:** 10.1186/s40850-021-00093-7

**Published:** 2021-10-07

**Authors:** Fatimat Tembotova, Ekaterina Kuchinova, Albina Amshokova, Ekaretina Kononenko

**Affiliations:** Tembotov Institute of Ecology of Mountain Territories of Russian Academy of Sciences, Nalchik, Russia

**Keywords:** Species diversity, *Mus macedonicus*, *Mus musculus*, Cytochrome *b*, Genetic diversity, The eastern part of the North Caucasus

## Abstract

**Background:**

There are two species of *Mus* in the Caucasus: *M. musculus* and *M. macedonicus*. *M. musculus* is widespread in the Caucasus, where the species is found everywhere from the Black to the Caspian Sea. *M. macedonicus* is ubiquitous Transcaucasia. The most north-astern border of its distribution in the Caucasus, according to the literature, is located in the Derbent region, near the border between Dagestan and Azerbaijan.

**Results:**

Cytochrome *b* mt-DNA of genus *Mus* research in this study in the Eastern Caucasus. About 70% of *M. musculus* haplotypes from the lowlands of Dagestan were recorded for the first time. One of these haplotypes accounts for approximately 25% of the total species diversity of haplotypes. *M. macedonicus* was found in only one locality, the Sarykum barchans, where this species prevails in number and accounts for 70% of the total number mice of the genus *Mus*. The species is characterized by low values of genetic diversity and nucleotide variability, which may indicate that the population originated from a small number of founders and may explain its relative isolation from the main range. The dating of the appearance of the ancestors of *M. musculus* in the east of the Russian Caucasus corresponds to 99-66 thousand years ago (at a mutation rate of 3-10% per million years).

**Conclusion:**

The results obtained suggest that the history of the appearance of *M. musculus* in the Eastern Caucasus is more ancient and is not associated with human agricultural activities.

We believe that possibly the ancestral range of *M. musculus* covered the eastern and western coasts of the Caspian Sea in the territory of southern Dagestan, Azerbaijan, and Iran. In this paper *M. macedonicus*, a Balkan-Asia Minor species, was registered for the first time in the North Caucasus. This species was registered in the center of Dagestan, where it inhabits sympatrically (on the territory) and syntopically (on the same biotope) with *M. musculus*. The low values of genetic diversity of *M. macedonicus* in the North Caucasus suggest that the population originated from a small group of founders.

## Background

The Caucasus, located at the junction of Europe and Asia, plays a very important role in the formation of the climate, natural ecosystems and their components [1, 2]. The interpenetration of flora and fauna taxa occurs across the Caucasian Isthmus. European taxa predominate in the North Caucasus; in Transcaucasia, representatives of the Near-Asian and Mediterranean faunas predominate.

Two species of *Mus* live in the Caucasus: *Mus musculus* (Linnaeus 1758) and *Mus macedonicus* (Petrov and Ruzic 1983). *M. musculus* has a wide distribution in the North Caucasus, where the species is found everywhere from the Black Sea coast to the Caspian Sea coast [3–5] and in the mountains up to 2000 m above sea level. Studies have shown [6, 7] that in Transcaucasia hybridization occurs between two taxa of house mice: *M. m. musculus* and *M. m. domesticus*. The wild species *M. macedonicus* also lives in the Transcaucasus.

*M. macedonicus*, a wild mouse living in the Balkans and Asia Minor, is distributed throughout Transcaucasia [6, 8–15]. This species is widespread in Adzharia [16], in north-east Georgia [6, 17–19], throughout most of Armenia (except the high mountains in the central regions), in valleys in the middle courses of the Araks River, Kura River and Alazani River, and in the western and north-western parts of Azerbaijan [4, 20]. The north-eastern border of the species range in the Caucasus is in Azerbaijan, where it runs along the southern slopes of the Greater (Main) Caucasus Ridge (Zkataly–Sheki) according to Kotenkova [4]. These data were verified by Macholán et al. [19], who found *M. macedonicus* in the vicinity of Derbent near the Dagestan border with Azerbaijan.

These two *Mus* species are known as twin species and do not differ in their morphological characteristics. However, different authors have still studied the distribution of *Mus* throughout most of the range without the use of molecular genetic methods.

We aimed to study the diversity of the genus *Mus* in the Eastern Caucasus within the Dagestan Nature Reserve based on mtDNA cytochrome *b* data.

## Results

We obtained mtDNA cytochrome *b* gene sequences of 549 bp for 50 specimens of the genus *Mus*, which correspond to positions 70–619 of the complete gene sequence. The *Mus* - identified haplotypes obtained in this study and taken from GenBank (https://www.ncbi.nlm.nih.gov/genbank/) are shown in Table 1. The haplotypes obtained in this study were deposited in GenBank under accession numbers MT036344-MT036364.

**Table 1:** Identified haplotypes the genus *Mus*

Haplotype	Species	Identification number	Collection point
1	2	3	4
1	*M. m. musculus*	MT036347^a^	Eastern part of North Caucasus
		AB649511^b^, AB649518^b^, AB649530^b^, AB649531^b^, AB649534^b^, AB649547^b^, KF839611^b^, KF839612^b^, KF697039^b^, MG748213^b^, MG748214^b^	Kazakhstan, Russia (Irkutsk), various provinces of China
2	*M. m. musculus*	MT036348^a^	Eastern part of North Caucasus
		AB649508^b^, AB649509^b^, AB649515^b^, AB819917^b^, AB649521^b^, KF839627^b^, KF697034^b^, KF697035^b^, KF697042^b^, KF697043^b^, KF697056^b^, KF697058^b^,	Poland, Hungary, Bulgaria, Ukraina, Moldova, Kazakhstan, Russia (Astrakhan, Kazan, Samara Region, Karelia)
3	*M. m. musculus*	MT036349^a^	Eastern part of North Caucasus
		AB649507^b^, AB649517^b^, AB649529^b^, AB649536^b^-AB649539^b^, AB649543^b^, AB649545^b^, AB649552^b^, KF839629^b^, KF697067^b^, AY057804^b^, KT376856^b^, KT376857^b^	Slovakia, Czech Republic, Estonia, Kazakhstan, Iran, Russia (Sulak), various provinces of China
4	*M. m. musculus*	MT036350^a^	Eastern part of North Caucasus
5	*M. m. musculus*	MT036351^a^	Eastern part of North Caucasus
6	*M. m. musculus*	MT036352^a^	Eastern part of North Caucasus
7	*M. m. musculus*	MT036353^a^	Eastern part of North Caucasus
8	*M. m. musculus*	MT036354^a^	Eastern part of North Caucasus
		AB649520^b^, KF697053^b^	Rumania, Russia (Primorye)
9	*M. m. musculus*	MT036355^a^	Eastern part of North Caucasus
10	*M. m. musculus*	MT036356^a^	Eastern part of North Caucasus
11	*M. m. musculus*	MT036357^a^	Eastern part of North Caucasus
12	*M. m. musculus*	MT036358^a^	Eastern part of North Caucasus
13	*M. m. musculus*	MT036359^a^	Eastern part of North Caucasus
		KF697037^b^	Russia (Moscow)
14	*M. m. musculus*	MT036360^a^	Eastern part of North Caucasus
15	*M. m. musculus*	MT036361^a^	Eastern part of North Caucasus
16	*M. m. musculus*	MT036362^a^	Eastern part of North Caucasus
17	*M. m. musculus*	MT036363^a^	Eastern part of North Caucasus
		KF697036^b^	Russia (Rostov region)
18	*M. m. musculus*	MT036364^a^	Eastern part of North Caucasus
19	*M. m. musculus*	AB649510^b^	Afghanistan
20	*M. m. musculus*	AB649512^b^	Uzbekistan
21	*M. m. musculus*	AB649513^b^	Uzbekistan
22	*M. m. musculus*	KT376858- KT376860^b^	Various provinces of Iran
		KT376874^b^	
23	*M. m. musculus*	KT376867-KT376869^b^	
24	*M. m. musculus*	KT376865-KT376866^b^	
25	*M. m. musculus*	KT376861-KT376863^b^	
26	*M. m. musculus*	KT376871^b^	Iran (Gorgan)
27	*M. m. musculus*	KT376873^b^	Iran (Mashhad)
28	*M. m. musculus*	KT376872^b^	Iran (Bojnord)
29	*M. m. musculus*	KT376870^b^	Iran (Bojnord)
30	*M. m. musculus*	KT376855^b^	Iran (Bojnord)
31	*M. m. musculus*	KT376854^b^	Iran (Bojnord)
32	*M. m. domesticus*	AY332705^b^	Switzerland
33	*M. m. domesticus*	KT376845-KT376847^b^, KT376849-KT376852^b^	Various provinces of Iran
34	*M. m. domesticus*	AB649479^b^	Various provinces of Iran
		KT376840-844^b^	
35	*M. m. domesticus*	KT376848^b^	Iran (Kordestan)
36	*M. m. domesticus*	KT376853^b^	Iran (Urmia)
37	*M. m. domesticus*	KX790793^b^	Pakistan (Attock)
38	*M. m. bactrianus*	KT376793^b^	Iran (Zahedan)
39	*M. m. bactrianus*	KT376773^b^	Afghanistan (Kabol)
40	*M. m. bactrianus*	KT376753^b^	Iran (Saravan)
41	*M. m. bactrianus*	KT376746^b^	Iran (Saravan)
42	*M. m. bactrianus*	KY661853^b^	Pakistan
43	*M. m. bactrianus*	KY661852^b^	
44	*M. m. bactrianus*	KY661851^b^	
45	*M. m. bactrianus*	KY661850^b^	
46	*M. m. bactrianus*	KY661846^b^	
47	*M. m. bactrianus*	KY661845^b^	
48	*M. m. bactrianus*	KY661836^b^	
49	*M. m. bactrianus*	KY661837-839^b^	
50	*M. m. bactrianus*	KY661840^b^	
51	*M. m. bactrianus*	KY661841^b^	
52	*M. m. bactrianus*	KY661831- KY661835^b^ KY661842- KY661844^b^, KY661847^b^, KY661854- KY661856^b^	
53	*M. m. bactrianus*	AB649490^b^	India (Delhi)
54	*M. m. castaneus*	KY418175^b^	Nepal (Lumbini)
55	*M. m. castaneus*	KY418176^b^	Nepal (Lumbini)
56	*M. m. castaneus*	AB819912^b^	India (Ghaziabad)
57	*M. m. castaneus*	AB819908^b^	India (Delhi)
		AB819909^b^	Various provinces of India
58	*M. m. castaneus*	AB819911^b^, AB820897 ^b^, KY418172^b^, KY418177^b^	Nepal (Lumbini)
59	*M. m. castaneus*	KY418173^b^	Nepal (Lumbini)
60	*M. m. castaneus*	AB649491^b^	Various provinces of India
		AB820901^b^, AB820910^b^, AB973114-116^b^, KY418174^b^	Nepal (Lumbini)
61	*M. macedonicus*	MT036344^a^	Eastern part of North Caucasus
62	*M. macedonicus*	MT036345^a^	Eastern part of North Caucasus
63	*M. macedonicus*	MT036346^a^	Eastern part of North Caucasus
64	*M. macedonicus*	AB125770^b^	Israel
65	*M. macedonicus*	AY057808^b^	Former Yugoslavia
66	*M. spicilegus*	AB125775^b^	Bulgaria
67	*M. spicilegus*	KY754048^b^	Austria
68	*M. spretus*	AB033700^b^	Unknown

^a^Own data

^b^GenBank Accession No (https://www.ncbi.nlm.nih.gov/genbank/)

 The average nucleotide composition of the studied fragment was 28.9% adenine, 29.6% thymine, 26.0% cytosine and 15.5% guanine. Twenty-one haplotypes were obtained; 15 of these were registered for the first time (Table 1).

**Fig. 1:**
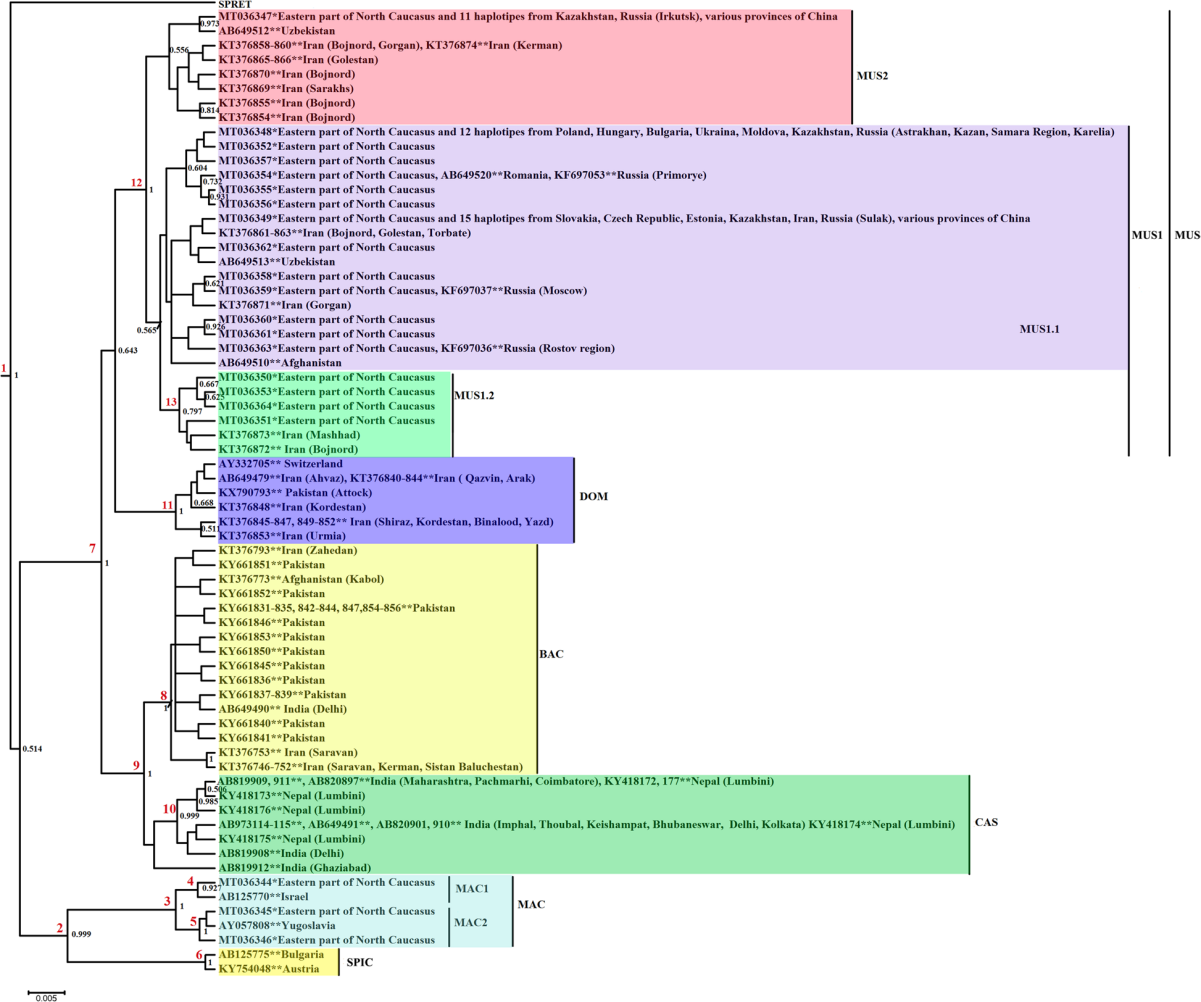
Cladogram of relationships between species of the genus *Mus* in the Eastern Caucasus from a cytochrome *b* gene fragment of 549 bp retrieved from GenBank with the Bayesian method. The numbers at the nodes indicate the posterior probabilities. Support values exceeding 0.5 are shown. * Data from the present study; ** GenBank Accession No. Abbreviations: SPIC – *M. spicilegus*; MAC – *M. macedonicus*; MAC 1 – MT036344 + Israel; MAC 2 – MT036345 + Yugoslavia; CAS – *M. m. castaneus*; BAC – *M. m. bactrianus*; MUS – *M. m. musculus*; DOM – *M. m. domesticus*; MUS 1 – *M. m. musculus* 1; MUS 2 – *M. m. musculus* 2; MUS 1.1 – *M. m. musculus* 1.1; MUS 1.2. – *M. m. musculus* 1.2; SPRET – *M. spretus*

Eighteen haplotypes obtained in this study were attributed to *M. musculus*, and three other haplotypes were attributed to *M. macedonicus* (Fig. 1). To interpret the results, a Bayesian tree was used (Fig. 1).

The dendrogram includes three well-differentiated haplogroups corresponding to three species of the genus *Mus*: *M. musculus, M. macedonicus,* and *M. spicilegus*, which is consistent with literature data [15]. Haplogroups in this tree are distinguished with a sufficiently high reliability and have large support (posterior probabilities of 0.514 and more). The outer group is *Mus spretus*.

The average genetic distance between haplotypes of these three species was as follows: 6.80% between *М. musculus* and *М. macedonicus*, 7.00% between *М. musculus* and *М. spicilegus*, and 5.23% between *M. macedonicus* and *M. spicilegus* (Table 2). The distance in three comparison options: *M. spretus* − *M. musculus*, *M. spretus* − *M. spicilegus*, and *M. spretus* − *M. macedonicus* is 10% or more.

**Table 2:** Interspecies genetic distances (*d*, %) of genus *Mus* for a 549-bp cytochrome *b* gene region

Species	*M. musculus*	*M. macedonicus*	*M. spicilegus*	*M. spretus*
*M. musculus* (*n* = 60)	–	1,00	1,06	1,46
*M. macedonicus* (*n* = 5)	6,8	–	0,95	1,55
*M. spicilegus* (*n* = 2)	7,0	5,2	–	1,48
*M. spretus* (*n* = 1)	11,4	11,0	10,2	–
Overall groups 4, *n* = 68				

Distance bottom left, standard error top right

The average intraspecies distances were approximately 10 times smaller; *d* (K2P) was 0.69% for *М. musculus* and 0.59% for *М. macedonicus* (Table 3).

**Table 3:** Average genetic distances (*d, %*) between mice of genus *Mus* populations*,* Kimura 2-parameter (K2P), calculated for 549-bp participation in cytochrome *b*

Species	*d*	S.E.
*M. musculus*	0.69	0.15
*M. macedonicus*	0.59	0.24

*S.E.* Standard error (%)

### Genetic differentiation of *M. musculus*

*M. musculus* has a distinct intraspecific structure (Fig. 1). In the basal part of the dendrogram, there are three subspecies with a high posterior probability: *M. m. castaneus*, *M. m. bactrianus* and *M. m. musculus*. Support *M. m. domesticus* is not so significant (0.643); nevertheless, this taxon is isolated and, probably, if the number of specimens increased, the support would be much higher. Cluster *M. m. musculus* is well structured; two clades are distinguished here, which correspond to those identified by Suzuki et al. [15] *M. m. musculus* 1 and *M. m. musculus* 2 (Fig. 1).

### *M. musculus* in the eastern part of the North Caucasus

All 18 haplotypes of *M. musculus* obtained in the eastern part of the North Caucasus fell into the cluster of one subspecies *M. m. musculus*. Seventeen haplotypes are located in the *M. m. musculus* 1, haplotype МТ036347 fell into the cluster *M. m. musculus* 2 (Fig. 1). Genetic distance between cluster *M. m. musculus* 1 and *M. m. musculus* 2 is 0.80%. Haplotype МТ036347 was identical to haplotypes from Kazakhstan, Uzbekistan, Russia (Irkutsk) and different regions of China and united in the cluster *M. m. musculus* 2 together with specimens from Iran. Subcluster *M. m. musculus* 1, which included 17 haplotypes from the eastern part of the North Caucasus, is distinguished by a great variety and structure. The two clades are well subdivided here, although the posterior probability has a low value of 0.565. Four haplotypes of the clade *M. m. musculus* 1.1 (MUS 1.1) are widespread in Europe (Poland, Hungary, Moldova, Ukraine, Czech Republic, Slovakia, Romania, Bulgaria, Estonia), the European part of Russia, Central Asia (Kazakhstan) and a number of regions of China. Haplotypes from Iran, Uzbekistan and Afghanistan fell into one clade. The second clade of *M. m. musculus* 1.2 (MUS 1.2) (posterior probability 0.797) includes two haplotypes from Iran and four haplotypes (МТ036350, МТ036351, МТ036353, МТ036364) from the eastern part of the North Caucasus. The distance between the two clades is 0.80%.

*M. m. musculus* in the eastern part of the North Caucasus is characterized by low values of nucleotide diversity (Table 4) and high values of haplotype diversity.

**Table 4:** Parameters of diversity of mtDNA haplotypes according to partial cytochrome *b* gene (549 bp) in the populations of the genus *Mus* in Eastern part of North Caucasus

Locality	N	Number of haplotypes, i	Nucleotide diversity π*,* %	Haplotypic diversity, H
*M. musculus*				
Sarykum barchans	3	3	0.607	1.000
Agrahansky	7	5	0.312	0.905
Kizlyar Bay	17	10	0.396	0.904
North-eastern border of the Republic of Dagestan and the Republic of Kalmykia	16	7	0.325	0.825
*M. macedonicus*				
Sarykum barchans	7	3	0.052	0.524

**Fig. 2:**
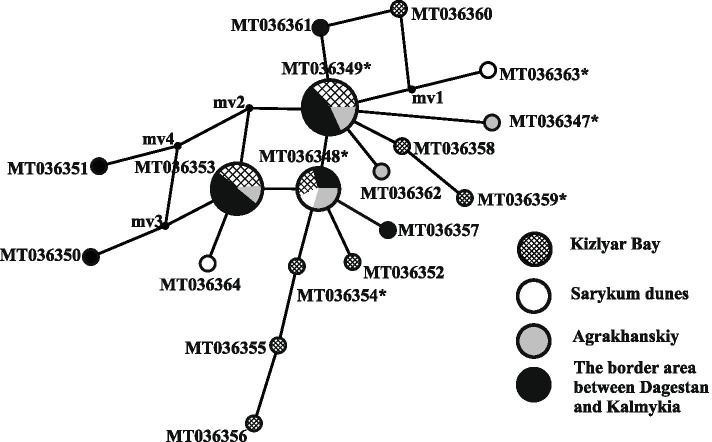
*M. musculus* haplotypes median-joining network. The length of the branches is 206 proportional to the number of mutations; the circle sizes are proportional to the number of 207 detected haplotypes. The numerals correspond haplotypes names, mv1–mv4 represent the 208 median vectors, hypothetical intermediate haplotypes that were not registered. The outside the 209 studied site variants are marked by asterisk (Table 1)

Twelve of the 18 *M. musculus* haplotypes obtained in the eastern part of the North Caucasus were registered for the first time (Fig. 2, Table 1). Three haplotypes (MT036348, MT036349, MT036353) were found the most often in the study area (Fig. 2).

Only one haplotype (MT036348) of *M. musculus* was obtained at all localities (Fig. 2); its share of the total sample was 16.3%. This haplotype is known from several European countries: Moldova and Bulgaria [15], Poland, Hungary, Ukraine [15, 21], the cities of Astrakhan and Kazan in Russia [15], Karelia and the Samara regions in Russia [21], and Kazakhstan (https://www.ncbi.nlm.nih.gov/genbank/). The share of the MT036349 haplotype was 25.6%; this haplotype was not found in the Sarykum barchans samples. Haplotype MT036349 is known from another region of Dagestan (Sulak) [15], as well as from other areas in Europe, namely, Slovakia [21], the Czech Republic [22], and Estonia [15]; it has also been found in Asia, in Kazakhstan (NCBI: KF839629), Iran [23] and in several provinces of China (Xiaogan, Laiyang, Shijiazhuang, Qiqihar, Baodi, Hohhot, Hulin) [15]. The haplotype MT036353 was registered to *M. musculus* for the first time. This haplotype had a high frequency; its share in the total sample was 23.3% in this study. The haplotype MT036353 differs from the MT036348 haplotype by one nucleotide substitution. Four haplotypes (MT036347, MT036354, MT036359, MT036363) were registered in the studied territory in single specimens of house mice, which are also known from Europe, the European part of Russia, and parts of Asia, specifically, Romania [21], Moscow [21], the Rostov region [21], Irkutsk [15], Kazakhstan ([15, 21], https://www.ncbi.nlm.nih.gov/genbank/), Primorsky Krai [15] and various provinces of China [15, 24]. Twelve haplotypes were found once and they are not in GenBank.

### Genetic differentiation of *M. macedonicus*

In this paper, *M. macedonicus*, a Balkan-Asia Minor species, was first recorded in the North Caucasus and in only one locality, the Sarykum barchans. Three haplotypes (MT036344-MT036346) were recorded among seven studied mice (Table 1) for the first time. The MT036345 haplotype was detected in 5 specimens, and the sequences MT036344 and MT036346, differing from MT036345 by 1–3 synonymous substitutions, were found in one specimen each. The average intraspecific distance for *M. macedonicus* in the eastern part of the North Caucasus was 0.49%. Three haplotypes were divided into two subclusters (Fig. 1). Haplotype MT036344 merged with a haplotype from Israel (MAC 1), MT036345 and MT036346 ended up in a subcluster with a haplotype from the former Yugoslavia (MAC 2). The distance between two subclusters (node 3) is 0.73%, which corresponds to the distance between two haplogroups *M. m. musculus* 1 and *M. m. musculus* 2 (0.78%).

### Time of divergence between *M. musculus* haplogroups and within them

In this study, we were unable to isolate the whole sequence of cytochrome *b* mtDNA; therefore, all the calculated dates are approximate. We will conduct more research in the future.

The divergence estimates obtained in BEAST are calculated for 5 variants of substitutions per million years: 3, 5, 10, 20, 40% (Table 5). Time estimates of 1.7 Ma for the root node divergence of *Mus spretus* and other species of the genus *Mus* were used as a calibration point, as suggested by Suzuki et al. [18]. The date of divergence of two haplogroups *M. m. musculus* 1 and *M. m. musculus* 2 (MUS 1 - MUS 2, 12 node) 150 ka ago [18]. According to our calculations, MUS 1 and MUS 2 diverged from a common ancestor ~ 185-131 thousand years ago, which corresponds to a mutation rate of 3-10% per million years ago. The age of the common ancestor of *M. m. musculus* 1.2 of the eastern Caucasus and related haplotypes from Iran dates back to 99-66 thousand years ago (node 13, Fig. 1, Table 5) for models calculated for a mutation rate of 3-10% per million years ago.

**Table 5:** Calculated divergence time of the nodes of the *Mus* dendrogram

Tree node number / substitution rate	3%	5%	10%	20%	40%
1 SPRET – 3SP	2.0332	1.8319	1.5881	1.3749	1.0884
2 MAC – SPIC	0.8064	0.737	0.6533	0.5782	0.4886
3 MAC	0.1233	0.1075	0.0882	0.0718	0.0586
4 MAC 1	0.0541	0.0474	0.0381	0.0311	0.0237
5 MAC 2	0.0345	0.0298	0.0246	0.0199	0.0163
6 SPIC	0.0287	0.0246	0.0209	0.0171	0.0137
7 CAS + BAC – MUS	0.4367	0.3945	0.3441	0.2985	0.2467
8 BAC	0.1072	0.0935	0.0746	0.0618	0.0485
9 BAC – CAS	0.2171	0.1918	0.1578	0.1316	0.1037
10 CAS	0.196	0.1725	0.1416	0.1172	0.0923
11 DOM	0.1022	0.0891	0.074	0.0596	0.0475
12 MUS 1 – MUS 2	0.1846	0.1611	0.1308	0.1049	0.0831
13 MUS 1.2	0.0991	0.085	0.0655	0.0571	0.0435

*Abbreviations*: *SPRET M. spretus*, *3SP M. macedonicus* + *M. spicilegus* + *M. musculus*, *MAC M. macedonicus*, *SPIC M. spicilegus*, *MAC 1* MT036344 + Israel, *MAC 2* MT036345 + Yugoslavia, *CAS M. m. castaneus*, *BAC M. m. bactrianus*, *MUS M. m. musculus*, *DOM M. m. domesticus*, *MUS 1 M. m. musculus* 1, *MUS 2 M. m. musculus* 2, *MUS 1.2 M. m. musculus* 1.2

### Time of divergence between *M. macedonicus* haplogroups

*M. macedonicus* and *M. spicilegus* diverged ~ 806-653 thousand years ago (mutation rate 3-10% per million years) (Table 5). The age of the *M. macedonicus* found in the Transcaucasia corresponds to ~ 123-88 thousand years ago (mutation rate is 3-10% per million years). The age of haplogroups MAC 1 and MAC 2 found in the Eastern Caucasus are ~ 54-38 thousand years and 35-25 thousand years, respectively, with a mutation rate of 3 -10% per million years (nodes 4-5, Fig. 1, Table 5).

## Discussion

We recorded *M. musculus* in all three sites under study in the Dagestansky Nature Reserve and around the northeastern border between the Republic of Dagestan and the Republic of Kalmykia. We applied Fst and Фst criteria to study the genetic differentiation of the *M. musculus* populations from 4 localities. The comparison of haplotype frequencies (Fst) shows the lack of significant differences; i.e., Fst values range from 2.40 to 7.74% at the significance level p (Fst) of 0.264–0.8896 (Table 6) between the populations.

**Table 6:** Frequency of occurrence of haplotypes (Fst) in the studied populations of *M. musculus*, calculated for 549-bp participation in cytochrome *b*

Locality	Sarykum barchans	Agrahansky	Kizlyar Bay	North-eastern border of the Republic of Dagestan and the Republic of Kalmykia
Sarykum barchans	–	0.7480	0.3453	0.2640
Agrahansky	− 0.0420	–	0.8350	0.7222
Kizlyar Bay	0.0240	−0.0451	–	0.8896
North-eastern border of the Republic of Dagestan and the Republic of Kalmykia	0.0774	−0.0382	−0.0317	–

At the bottom left are the estimates of Fst, at the top right the level of their significance

Pairwise Фst values varied from 0.14 to 2.04; the differences related to Фst values were also unreliable (Table 7).

**Table 7:** Pairwise estimates of genetic differentiation (Фst) between the studied populations of *M. musculus*, calculated from the 549-bp region of the cytochrome *b* gene

Locality	Sarykum barchans	Agrahansky	Kizlyar Bay	North-eastern border of the Republic of Dagestan and the Republic of Kalmykia
Sarykum barchans	–	0.4225	0.4782	0.4014
Agrahansky	− 0.0081	–	0.4443	0.3203
Kizlyar Bay	−0.0169	−0.0038	–	0.2088
North-eastern border of the Republic of Dagestan and the Republic of Kalmykia	−0.0014	0.0091	0.0204	–

The estimates of Фst are at the bottom-left; the levels of their significance are at the top-right

These results demonstrate that the four studied populations of house mice in the Eastern Caucasus are panmictic, with a high level of gene exchange between them. This is facilitated by the abundance of the species. *M. musculus*, in the four studied localities, represents 1.1-9.9% or more of the total abundance of small mammals (Rodentia, Eulipotyphla) [25, 26].

The registered 11 unique haplotypes and MT036353, found only in Dagestan, together accounted for about 70% of the haplotype diversity in the four house mice populations of the Eastern Caucasus. This indicates that the populations of *M. musculus* in eastern Dagestan are, to a certain extent, isolated from the populations of domestic mice in the European part of Russia. It can be assumed that haplotype MT036353 is endemic for *M. musculus* of eastern Dagestan since its frequency represents approximately 25% of the total diversity of haplotypes and it is found at the edge of the species range.

The results obtained in BEAST (Table 5) showed that the common ancestor of the clade *M. m. musculus* 1 and *M. m. musculus* 2 (12 node, Fig. 1) appeared on the territory of the eastern part of the North Caucasus ~ 185-131 thousand years ago (3-10% of substitutions per million years), but not earlier than ~ 83 thousand years (40% of substitutions per million years). In general, these dates are comparable to those of Suzuki et al. [15].

The common ancestor of the MUS 1.2 cluster separated from the main part of *M. m. musculus* 1 (node 13) within ~ 99-66 thousand years ago (3-10% of substitutions per million years) but not earlier than ~ 43 thousand years (40% of substitutions per million years) (Table 5).

Thus, it can be assumed that the common ancestor of *M. m. musculus* 1 appeared in the Caucasus ~ 185-131 thousand years ago in the Upper Pleistocene, and MUS 1.2 during the last ice age ~ 99-66 thousand years ago, but before LGM. These results are consistent with the data of N.K. Vereshchagin [27], who dated the paleontological material for *M. musculus* from foothill Dagestan to the Middle Pleistocene.

The results obtained suggest that the distribution of *M. musculus* in the Eastern Caucasus is not associated with human agricultural activity. We believe that, perhaps, the ancient range of *M. musculus* covered the eastern and western coasts of the Caspian Sea, in the eastern part of the Russian Caucasus, Azerbaijan and Iran.

We identified *M. macedonicus*, as opposed to the house mouse, only in one locality, i.e., ‘Sarykum barchans’, where the species was dominant in number and accounted for 70% of the total number of mice genus *Mus*. The vicinity of Makhachkala is known today as the northernmost border of the distribution of *M. macedonicus*. The species is characterized by low values of gene diversity and nucleotide variation (Table 2), which may indicate the origin of the population from a small number of founders and its relative isolation from the main range of the species. The identified clear tree structure of the species (Fig. 1) is probably also explained by the high level of ancestral polymorphism in the discovered population of *M. macedonicus*.

The common ancestor of the MAC 1 and MAC 2 subclusters (node 3) has an age of ~ 123-88 thousand years ago, which corresponds to a mutation rate of 3-10% per million years.

Age of haplogroup MAC 1 (node 4, Fig. 1, Table 5) corresponds to ~ 54-38 thousand years ago (mutation rate 3-10% per million years), haplogroup MAC 2 (node 5) corresponds to ~ 35-25 thousand years ago (mutation rate 3-10% per million years). This suggests the appearance of *M. macedonicus* in the Transcaucasia not later than in the Upper Pleistocene. Now it is difficult to conclude whether the haplotypes of *M. macedonicus* first recorded by us are unique and how long ago they appeared in the eastern part of the North Caucasus. Further researches are needed.

## Conclusion

Our study of the cytochrome *b* mtDNA gene region in mice of the genus *Mus* showed that two species inhabit the lowland of the eastern part of the North Caucasus: *M. musculus* and *M. macedonicus*. In this paper *M. macedonicus*, a Balkan-Asia Minor species, was registered for the first time in the North Caucasus.

*M. musculus* is widespread in the lowland Dagestan and its range covers various localities in which it is numerous or common. *M. macedonicus* is limited in its distribution to the southernmost point in the studied area of the Dagestan reserve near the town of Makhachkala. The populations of *M. musculus* in the Eastern Caucasus are panmictic, with a high level of gene exchange between them. Only about 30% of the Eastern Caucasus house mouse haplotype diversity is identical to the diversity of haplotypes from other areas of the species range in Eurasia, from Western Europe to the Far East. About 70% of haplotypes were registered for the first time which perhaps allows them to be considered endemic for the eastern part of the North Caucasus. This also indicates a high degree of isolation of the genetic diversity of *M. musculus* in the eastern part of the Caucasus from European diversity (Western and Eastern Europe, including the European part of Russia).

It is more likely that the dating of the appearance of the ancestors of *M. musculus* in the eastern Caucasus corresponds to one of three models calculated for 3-10% substitutions per million years, which allows us to propose a dating of ~ 185-131 thousand years, but not earlier than ~ 43 thousand years, i.e. to LGM.

The results obtained suggest that the distribution of *M. musculus* in the Eastern Caucasus is not associated with human agricultural activities. We believe that perhaps the ancestral range of *M. musculus* covered the eastern and western coasts of the Caspian Sea in the territory of southern Dagestan, Azerbaijan, and Iran.

The low values of the genetic and nucleotide diversity of the population of *M. macedonicus* from the Sarykum barchans region suggest the origin of this population from a small number of founder individuals as a result of natural or accidental introduction. It could be considered, the border of the expanding range of this species could be located anywhere in central Dagestan; to provide support for this view, further studies in the Eastern Caucasus are necessary.

*M. macedonicus* probably appeared in the Transcaucasia ~ 123-88 thousand years ago (mutation frequency 3-10% per million years), but not earlier than ~ 57 thousand years ago, which corresponds to the period of the Upper Pleistocene. The age of haplogroup MAC 1 corresponds to ~ 54-38 thousand years ago (mutation rate 3-10% per million years), haplogroup MAC 2 corresponds to ~ 35-25 thousand years ago (mutation frequency 3-10% per million years). Now it is difficult to conclude whether the haplotypes of *M. macedonicus* first recorded by us are unique and how long ago they appeared in the eastern part of the North Caucasus. Further researches are needed.

## Methods

### Study area

**Fig. 3:**
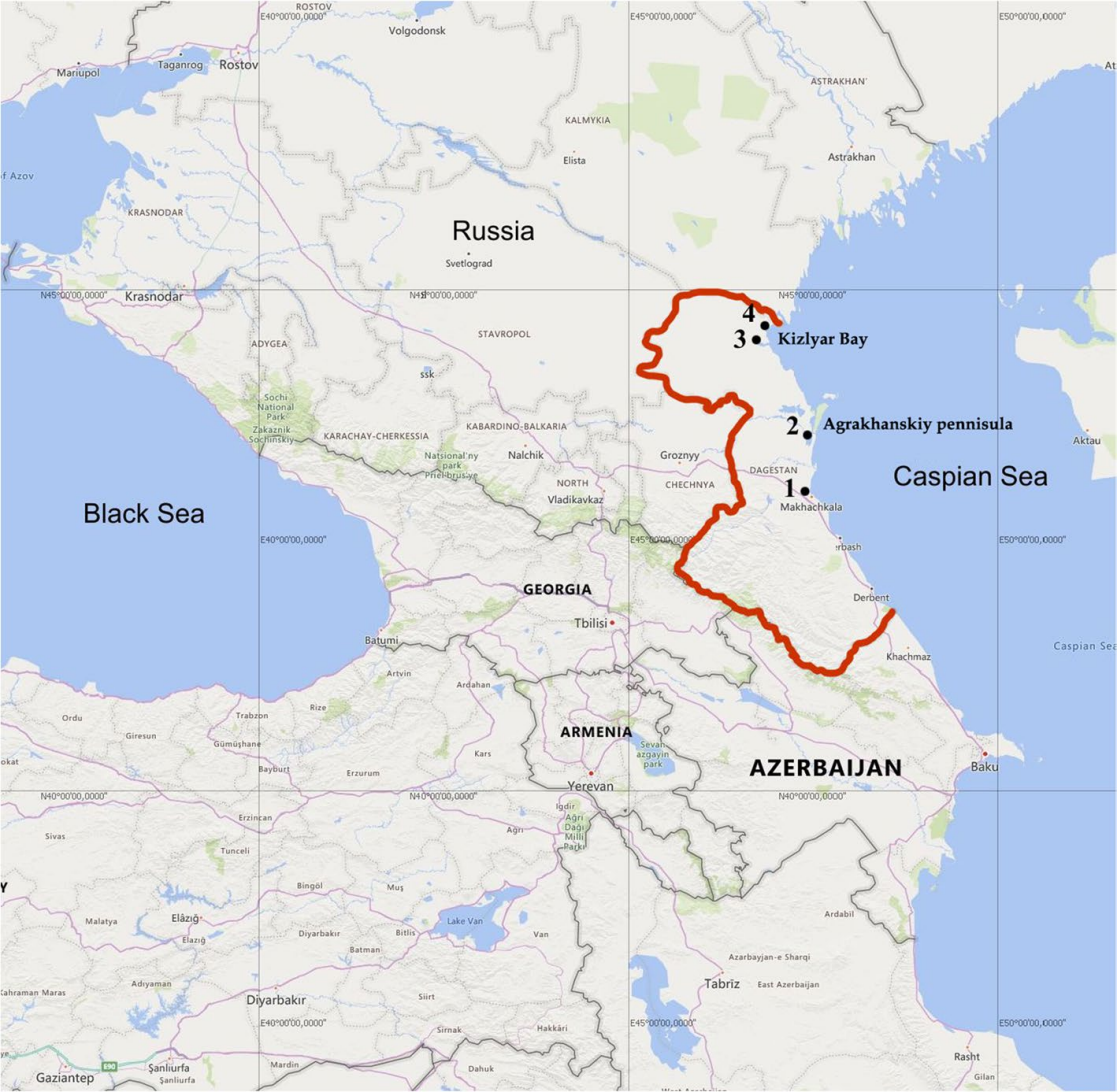
Map of the study area in the eastern part of the North Caucasus: 1–Sarykum barchans (geographical coordinates 43°00.472′ N, 47°13.808′ E), 2–Agrahansky (43°48.255′ N, 47°26.866′ E); 3–Kizlyar Bay (44°30.995′ N, 46°47.656′ E); 4–the border area between Dagestan and Kalmykia (44°46.452′ N, 46°57.900′ E). The red line indicate the Republic of Dagestan border. Open access Caucasus map used in the paper (“© OpenStreetMap contributors”. https://www.openstreetmap.org/copyright)

The research was carried out in the Eastern Caucasus in the Dagestan Reserve. The reserve is a protected area of 5 plots, 4 of which are located in the lowlands: Sarykum barchans (1.175 thousand hectares), Agrahansky (39.0 thousand hectares), Kizlyar Bay (land 9.185 thousand hectares), Samursky (11.200 thousand hectares). The natural landscapes of the reserve are distinguished by the high diversity and complexity of the ecosystems, the main types of which are sand barchans, dry foothills, delta forests and high mountains [28]. The studies were conducted in the lowland areas of the reserve: locality 1 – Sarykum barchans (geographical coordinates 43°00.472′ N, 47°13.808′ E; 100 m a.s.l.), locality 2 – Agrahansky (43°48.255′ N, 47°26.866′ E; 50 m a.s.l.), locality 3 – Kizlyar Bay (44°30.995′ N, 46°47.656′ E; 50 m a.s.l.), and locality 4 – the border area between Dagestan and Kalmykia (44°46.452′ N, 46°57.900′ E; 30 m a.s.l.) (Fig. 3). Distance between localities (km, in a straight line): 1–2 – 90 km; 1–3 – 175 km; 1–4 – 200 km.

A visual description studied biotopes of four localities is below. Grassy tiers were recorded in all localities. 1) The Sarykum barchans phytocenoses are represented by various forest-steppe areas. The first community of plants includes a tree tier of *Robinia pseudoacacia* with hybrid poplar, a shrub tier of *Tamarix ramosissima*. The second community of plants includes a wood tier of a few hybrid poplar trees and a shrub tier of *Elaeagnus caspica* and *T. ramosissima*. The third community of plants includes artificial plantings of *Ailanthus altissima* with an admixture of hawthorn and *Populus alba*. 2) The plant communities in Kizlyar Bay include the reed *Phragmites australis* and wormwood. 3) The locality of Agrahansky covers the territories of several plant communities. The biotope include a shrub tier, represented by islets of *T. ramosissima*. The second community includes a shrub layer of *T. ramosissima*. The third community includes a shrub tier of *T. ramosissima*. The fourth community is represented by a Tugai forest of *Morus* sp., *Amorpha fruticosa* and *Salix triandra*; the shrub tier is dominated by Rubus. 4) Plant communities on the border between the Republics of Dagestan and Kalmykia include the shrub tier, represented by *T. ramosissima* and *Tamarix gracilis*.

### Materials

In total, 50 wildlife mice of the genus *Mus* were studied: 10 specimens from locality 1, 7 specimens from locality 2, 17 specimens from locality 3 and 16 specimens from locality 4.

All *Mus* specimens were identified by body size, fur colouration and structure of the skull and teeth [2]. Mice were euthanized with 8% sevoflurane under special conditions, after which the animals were sacrificed by decapitation.

### Methods

Total DNA was extracted from muscle tissue and preserved in 96% ethanol using a Diatom™ DNA Prep 100 kit (Izogen Laboratory Ltd., Moscow, Russia) according to the manufacturer’s protocol. The resulting DNA solutions were stored at − 18°С. The amplification of fragments of the mitochondrial cytochrome *b* gene was conducted by MasterMix Х5 (Dialat Ltd., Moscow). We used primers L14115: GACATGAAAAATCATCGTTG and H15300: GTTTACAAGACCAGAGTAAT for PCR under the parameters outlined in Yasuda et al. [29]. The resulting PCR products were purified by precipitation in a 0.15 M CH3COONa solution in 90% ethanol and then rinsed with 70% ethanol. The products were visualized by 1.5% agarose gel electrophoresis with ethidium bromide.

Sequencing of the nucleotide sequences of the mtDNA cytochrome *b* gene region was performed according to Senger using the BigDye Terminator v3.1 commercial kit (ThermoFisher) and the ABI 3130xl genetic analyser (ThermoFisher) at Sintol CJSC (Moscow).

We aligned the resulting sequences using BioEdit 7.09.0 [30]. We determined similar sequences (haplotypes) using the online toolbox FaBox 1.5 [31]. The phylogenetic tree was built in the program in MrBayes 3.2.6 [32] for 1,000,000 iterations and 1000 iterations of burn in. We used the HKY with gamma distribution and invariant sites (HKY + G + I) model. We performed the determination of the appropriate model in MEGA 6 [33].

For the analyses we also retrieved homologous mtDNA haplotypes from GenBank of mice from the genus *Mus*: *М. musculus*; *М. macedonicu*s; *М. spicilegus* (Table 1). *M. spretus* was used as an outgroup taxa; the south-eastern border of the contemporary range of this species in European Russia is in the Rostov region, which borders the Western Caucasus.

We estimated the age of the most recent common ancestors (TMRCAs) for mtDNA clades in BEAST V1.10.4 [34] by the Bayesian Markov-chain Monte-Carlo (MCMC) method, using the HKY + G + I substitution model as selected in MEGA 6. We used strict clock as clock model and constants size as coalescent model. We took all the calibration points used in the estimations from Suzuki et al. [15]. For the analysis, we used *M. spretus* as outgroup taxa. We used as calibration point the time estimates of 1.7 mya for the root node of the divergence of *M. spretus* and the other species of *M. musculus* species group. We used as calibration point the time estimates of 0.914 mya for the root node of the divergence of *M. macedonicus* and *M. spicilegus*. At last, we used as calibration point the time estimates of 0.459 mya for the root node of the divergence of subspecies of *M. musculus*. We run MCMC for 10,000,000 iterations and 1000 iterations of burn in.

We used the ARLEQUIN V.3.11 package [35] to evaluate genetic variation within species samples by assessing the parameters for haplotypic diversity (H) [36] and nucleotide diversity (π) [37]; statistical estimates of the differences between the populations based on the frequency of haplotypes (Fst); values of the pairwise distances between nucleotide sequences in the samples (Φst); and the degree of statistical validity in the obtained differences. We constructed median-joining networks of haplotypes using the NETWORK v.4.6.1.1 package [38].

## Data Availability

The *Mus* haplotypes registered by us were deposited in the GenBank (https://www.ncbi.nlm.nih.gov/genbank/) MT036344- MT036364

## Abbreviations

mtDNA: Mitochondrial DNA
PCR: Polymerase chain reaction
NCBI: National Center for Biotechnology Information
K2P: Kimura2 Parameter
H: Haplotype diversity
π: Nucleotide diversity
bp: Base pair
Fst: Frequency of haplotypes
Фst: Phylogenetic proximity of all haplotypes
